# The complete chloroplast genome of *Taraxacum albidum* (Asteraceae), a Japanese endemic dandelion

**DOI:** 10.1080/23802359.2024.2387258

**Published:** 2024-08-07

**Authors:** Haruka Mouri, Mizuki Tatsumi, Takako Nishino, Takeshi Suzuki, Tatsuyoshi Morita, Motomi Ito, Takaya Iwasaki

**Affiliations:** a Graduate School of Humanities and Sciences, Ochanomizu University, Bunkyo-ku, Japan; b Regional Environmental Planning Inc., Sapporo-shi, Japan; c Graduate School of Science, Osaka Metropolitan University, Sakai, Japan; d Institute of Natural and Environmental Sciences, University of Hyogo, Sanda, Japan; e Faculty of Education, Niigata University, Niigata, Japan; f Graduate School of Arts and Sciences, University of Tokyo, Meguro-ku, Japan

**Keywords:** Chloroplast genome, Asteraceae, *Taraxacum albidum*, dandelion, Japan

## Abstract

*Taraxacum albidum*, a perennial herb of the Asteraceae family, exhibits both tetraploid and pentaploid in Japan. This study sequenced and characterized the complete chloroplast genome of *T. albidum*, revealing a 151,451 bp sequence with a typical quadripartite structure, comprising one large single-copy (LSC) region of 84,052 bp, one small single-copy (SSC) region of 18,541 bp, and two inverted repeat (IR) regions, IRa and IRb, each 24,429 bp in length. The chloroplast genome, excluding duplicates, contained 113 unique genes, including 79 protein-coding genes, 30 transfer RNA genes, and four ribosomal RNA genes. The GC content of this genome was 37.7%. Phylogenetic analysis revealed that *T. albidum* is most closely related to *T. mongolicum*, with the chloroplast genome sequences being nearly identical, differing by only one nucleotide. These findings suggest that the maternal lineage of *T. albidum* likely originates from *T. mongolicum* or its closely related species.

## Introduction

**Figure 1.**
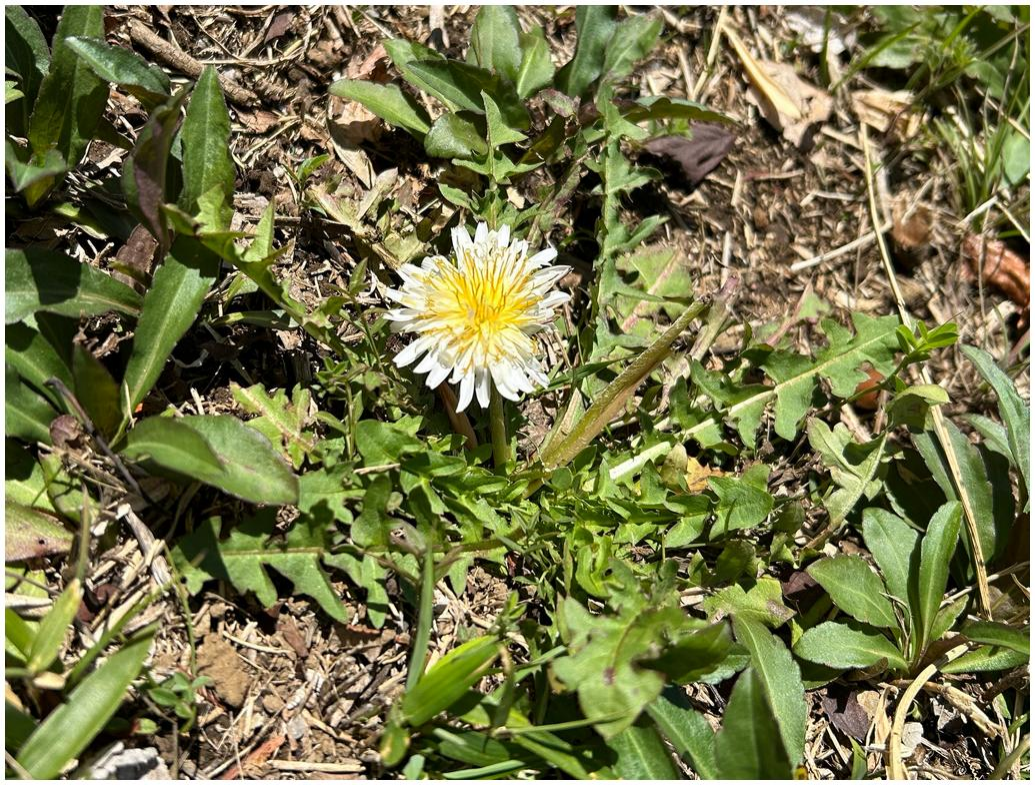
Image of *Taraxacum albidum* in bloom. This species is a perennial herb with white flowers and oblanceolate to linear-lanceolate leaves that are pinnatilobed or pinnatifid. The lateral lobes are triangular, either entire or irregularly dentate, often dentate between the lobes. The terminal lobe is also triangular and ranges from obtuse to acute at the apex. This photograph was taken by the first author Haruka Mouri on 14 February 2024, at Ochanomizu University, Tokyo, Japan. It is provided here free of any copyright restrictions.

The genus *Taraxacum* Weber ex F.H.Wigg. 1780 (Asteraceae), known as dandelion, exhibits a high species diversity, comprising 2516 species (WFO 2023). In Japan, 14 species, one subspecies, and two varieties are distributed (Kadota et al. 2017). Although molecular phylogenetic analysis of this genus *Taraxacum* has been conducted using intergenic regions of chloroplast DNA and nuclear ITS regions (Wittzell 1999; Kirschner et al. 2015), the evolutionary relationships remain insufficiently resolved due to the complexity of genome composition resulting from multiple hybridizations and genome duplications. Additionally, since only a few Japanese species have been included in the previous studies, the phylogenetic relationships among Japanese dandelions are still unclear. We focused on *T. albidum* Dahlst. 1907 (Figure 1), an endemic and agamospermous species of the Japanese archipelago (Morita 1995). This species is known to exhibit both tetraploid (2*n* = 32) and pentaploid (2*n* = 40). A detailed cytogeographical study surveying 1308 individuals from over 200 localities in Japan found tetraploid individuals are rare and confined to Kyushu, while pentaploid individuals are widespread across the entire range (Sato et al. 2011). Based on the distribution, we presume the individual collected in Tokyo for this study to be pentaploid. The objective of this study is to construct the complete chloroplast genome of *T. albidum* and elucidate its maternal lineage, presumed to have originated from past hybridization events.

## Materials and methods

The plant material of *T. albidum* was collected at Ochanomizu University, Bunkyo-ku, Tokyo, Japan (latitude 35.7221° N, and longitude 139.7401° E) according to local regulations and with permission. The specimen was deposited in the Makino Herbarium of Tokyo Metropolitan University (Herbarium code: MAK) (https://www.biol.se.tmu.ac.jp/herbarium/) (contact person: Noriaki Murakami, nmurak@tmu.ac.jp) under the specific identifying voucher number MAK-470661. Total genomic DNA was extracted from the dried leaf tissue of *T. albidum* using the Wizard Genomic DNA Purification Kit from Promega (Madison, WI). Prior to the standard DNA extraction procedure of the kit, we conducted two separate washes using PVP-HEPES buffer (pH 8.0 HEPES buffer, polyvinylpyrrolidone (PVP), l-ascorbic acid, 2-mercaptoethanol) (Setoguchi and Ohba 1995). PVP adsorbs polyphenols, thus inhibiting DNA-polyphenol binding (Echevarría-Machado et al. 2005). The extracted DNA was subcontracted to BGI (16 Dai Fu Street, Tai Po Industrial Estate, Tai Po, New Territories, Hong Kong) for shotgun sequencing with a data volume of 5 GB, NGS platform DNBSEQ, and read length PE 150. Adapter trimming and quality filtering were conducted using the software fastp v.0.20.0 (Chen et al. 2018) with default settings. *De novo* assembly of the chloroplast genome was conducted using NOVOPlasty v. 4.3.1 (Dierckxsens et al. 2017) (type: chloro, genome range: 120,000–180,000, K-mer: 39). The chloroplast genome sequence of *T. mongolicum* Hand.-Mazz. 1907 (GenBank accession number KU736961) (Kim, Park, Lee, Woo, et al. 2016) was used as a seed sequence for the NOVOPlasty analysis. To validate the accuracy of the assembly, we mapped trimmed reads to the assembled chloroplast genome and evaluated the depth of coverage using python scripts (Ni et al. 2023). Annotation of the obtained chloroplast genome was carried out using the software GeSeq (Tillich et al. 2017) and CPGAVAS2 (Shi et al. 2019) to identify protein-coding genes, tRNAs, and rRNAs. When discrepancies arose between the annotations provided by GeSeq and CPGAVAS2, manual corrections were made, guided by the annotation results from other species in the genus *Taraxacum*. The *de novo* assembly resulted in two patterns of chloroplast genome with full lengths; the difference between the two sequences was whether the SSC sequence is inverted. Both the two chloroplast genome sequences of *T. albidum* were annotated, and we selected one with homologous aligned sequences with that of *T. mongolicum*. The CPGView (Liu et al. 2023, http://www.1kmpg.cn/cpgview/) was used to draw the structural characteristics of the chloroplast genome and visualize the intron-containing genes.

To confirm the phylogenetic position of *T. albidum*, 10 complete chloroplast genome sequences of other *Taraxacum* species were downloaded from GenBank, and *Ixeris repens* A.Gray 1858 (Asteraceae) was used as outgroup. Because SSC sequences were inverted in two species (*T. obtusifrons* Markl. 1940 and *T. amplum* Markl. 1940), they were inverted and oriented before the phylogenetic analysis. The chloroplast genome sequences were aligned using MAFFT v. 7.490 (Katoh and Standley 2013) with default settings in the software Geneious Prime 2022 (Biomatters Ltd., Auckland, New Zealand). A maximum-likelihood phylogenetic tree was constructed using RAxML v. 8 (Stamatakis 2014) in Geneious Prime. The GTRGAMMA model was used for the nucleotide substitution model, and 1000 bootstrap replicates were carried out.

## Results

**Figure 2.**
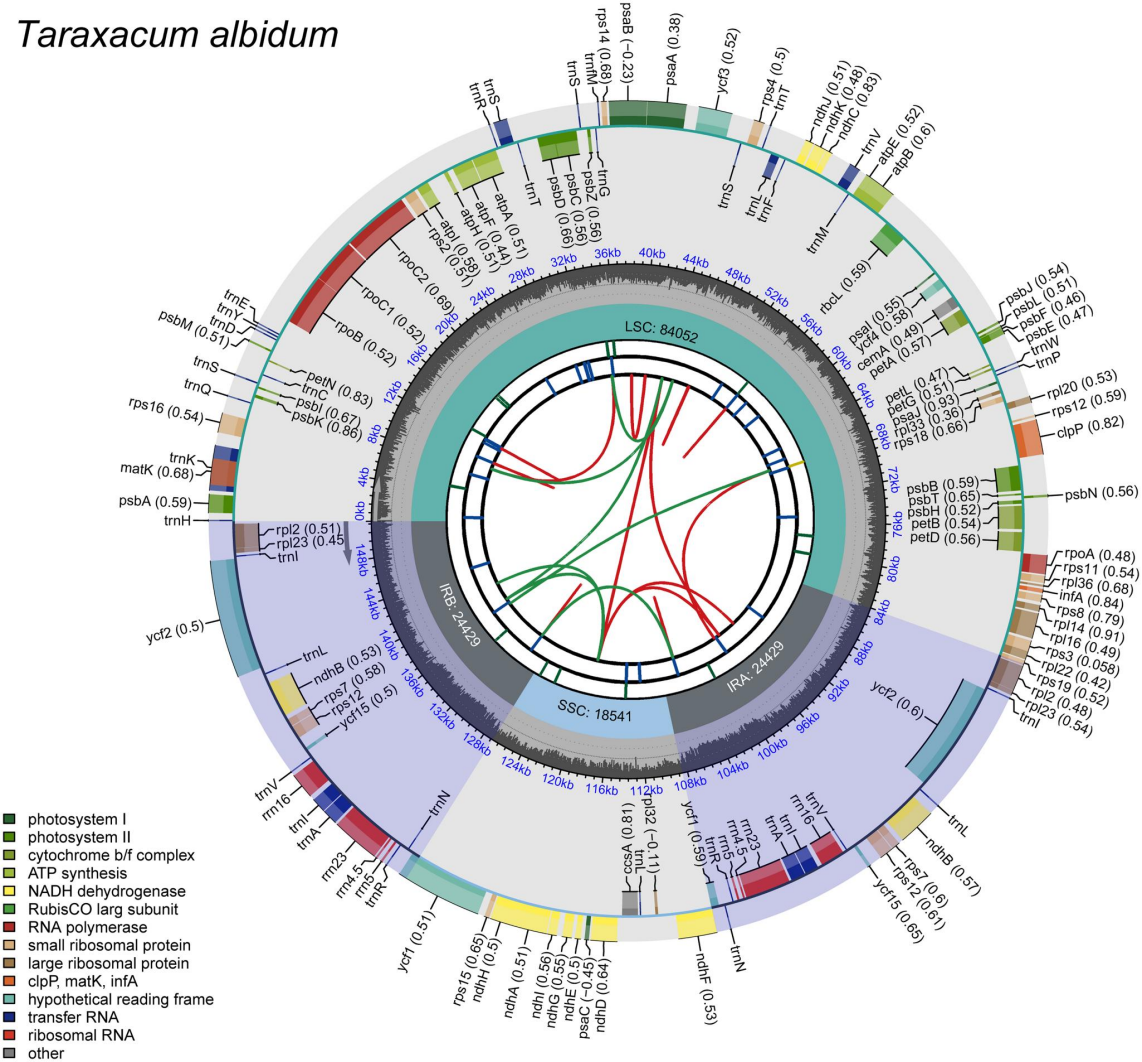
Complete chloroplast genome map of *Taraxacum albidum*. This circular map, generated by CPGView (http://www.1kmpg.cn/cpgview/), illustrates the chloroplast genome’s structure across six concentric tracks. Starting from the center, the first track shows the dispersed repeats, including direct (D) and palindromic (P) repeats, linked by red and green arcs, respectively. The second track depicts long tandem repeats as short blue bars, while the third track visualizes short tandem repeats (or microsatellites) with color-coded bars, indicating different repeat types. The fourth track delineates the genome’s regions: the small single-copy (SSC), large single-copy (LSC), and inverted repeats (IRa and IRb). The fifth track plots the GC content variation across the genome. The outermost track displays genes, color-coded according to function, with gene transcription directions indicated by the orientation: clockwise for inner genes and counterclockwise for outer genes. Codon usage bias, where applicable, is noted in parentheses following gene names. A legend in the bottom left clarifies the functional classification of genes by color.

**Figure 3.**
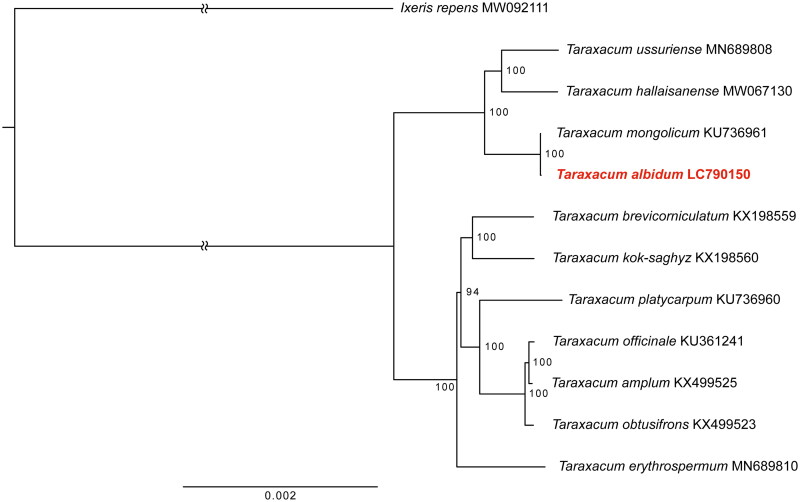
Maximum-likelihood (ML) phylogenetic tree constructed from complete chloroplast genome sequences of 11 *Taraxacum* species and *Ixeris repens* as an outgroup. Bootstrap values, based on 1000 iterations, are shown at each node to indicate confidence levels. The species in this study is highlighted in red. The chloroplast genome sequences analyzed, with their respective GenBank accession numbers, include: *Ixeris repens* MW092111 (Lee, Yang, Kim, et al. 2021), *Taraxacum ussuriense* Komarov 1932 MN689808 (Lee, Kim, Woo, et al. 2021), *Taraxacum hallaisanense* Nakai 1915 MW067130 (Lee, Kim, Kim, et al. 2021), *Taraxacum mongolicum* KU736961 (Kim, Park, Lee, Woo, et al. 2016), *Taraxacum brevicorniculatum* Korol. 1940 KX198559 (Zhang et al. 2017), *Taraxacum kok-saghyz* Rodin 1933 KX198560 (Zhang et al. 2017), *Taraxacum platycarpum* Dahlst. 1907 KU736960 (Kim, Park, Lee, Woo, et al. 2016), *Taraxacum officinale* F.H.Wigg. 1780 KU361241 (Kim, Park, Lee, Lee, et al. 2016), *Taraxacum amplum* KX499525 (Salih et al. 2017), *Taraxacum obtusifrons* KX499523 (Salih et al. 2017), and *Taraxacum erythrospermum* Andrz. ex Besser 1822 MN689810 (Lee, Kim, Woo, et al. 2021).

From the shotgun sequencing, we obtained 20,073,031 raw reads, with 96.15% of them having a Q20 quality score or higher. The assembled complete chloroplast genome of *T. albidum*, with a GenBank accession number of LC790150, was 151,451 bp in length, had an overall GC content of 37.7%, and an average coverage of ×908.33 (Figure 2 and Supplementary Figure 1). It exhibited a typical quadripartite structure comprising one large single-copy (LSC) region of 84,052 bp, one small single-copy (SSC) region of 18,541 bp, and two inverted repeat (IR) regions, IRa and IRb, each 24,429 bp in length (Figure 2). After excluding duplicates, the chloroplast genome included 113 unique genes: 79 protein-coding genes, 30 transfer RNA genes, and four ribosomal RNA genes. Among these, eight protein-coding genes, seven transfer RNA genes, and all four ribosomal RNA genes were duplicated, with each gene represented in both the IRa and IRb regions. Thirteen protein-coding genes were cis-spliced, with 11 of them (*rps16*, *rpoC1*, *atpF*, *petB*, *petD*, *rpoA*, *rpl16*, *rpl2*, *ycf2*, *ndhB*, and *ndhA*) containing a single intron, and two (*clpP* and *ycf3*) containing two introns each (Supplementary Figure 2A). Additionally, the placement of exon regions in the trans-splicing gene *rps12* was identified (Supplementary Figure 2B). In the constructed phylogenetic tree, *T. albidum* was most closely related to *T. mongolicum*, a triploid species distributed from Mongolia to the Korean Peninsula and Japan (Figure 3). The chloroplast genome sequences of *T. albidum* and *T. mongolicum* were nearly identical, showing 99.999% similarity with only one nucleotide difference.

## Discussion and conclusions

Since chloroplasts are predominantly maternally inherited in most angiosperms (Hagemann 2004), the phylogenetic tree based on chloroplast genomes provide insights into the maternal lineage. These results suggest that the maternal lineage of *T. albidum* is likely *T. mongolicum*. Based on allozyme analysis results, Morita et al. (2021) proposed a hybrid origin of the pentaploid *T. albidum* from an unreduced gamete of the tetraploid *T. albidum* and a reduced gamete of any diploid *Taraxacum* species. This hypothesis aligns with our findings. Combining this hypothesis and our results, we infer that the direct maternal species of the pentaploid *T. albidum* is the tetraploid *T. albidum*, and that the maternal species of the tetraploid *T. albidum* is *T. mongolicum*. However, our study did not encompass all *Taraxacum* species within this geographical range, so there might be species more closely related to *T. albidum*. The continued collection and analysis of chloroplast genome data from various species within this genus will further elucidate the detailed evolutionary history.

## Supplementary Material

SupplymentaryFigure_2 600dpi6inch.tif

SupplymentaryFigure_1 600dpi6inch.tif

## Data Availability

The genome sequence data supporting this study’s findings are available at the DNA Data Bank of Japan (DDBJ) Center (http://www.ddbj.nig.ac.jp DDBJ) under accession numbers LC790150. The associated BioProject, SRA, and Bio-Sample numbers are PRJDB15494, DRR452812, and SAMD00588590, respectively.
