# Supplementary figures and images for: The complete chloroplast genome of Taraxacum albidum (Asteraceae), a Japanese endemic dandelion
Source: Mitochondrial DNA B Resour. 2024 Aug 7;9(8):1015–9. doi: 10.1080/23802359.2024.2387258 (PMC11308969; doi:10.1080/23802359.2024.2387258)

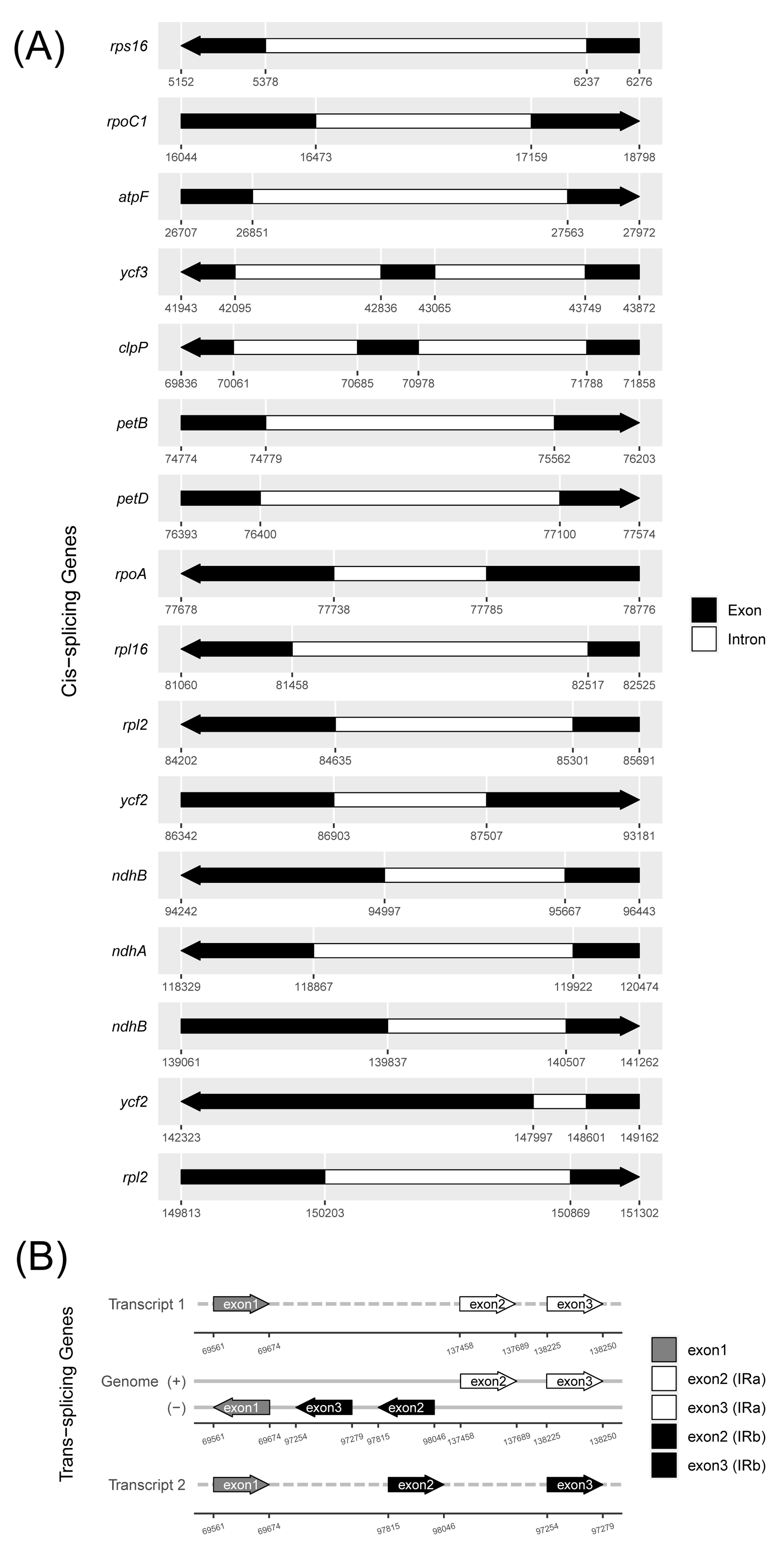

Supplement: SupplymentaryFigure_2 600dpi6inch.tif [file TMDN_A_2387258_SM4779.tif]

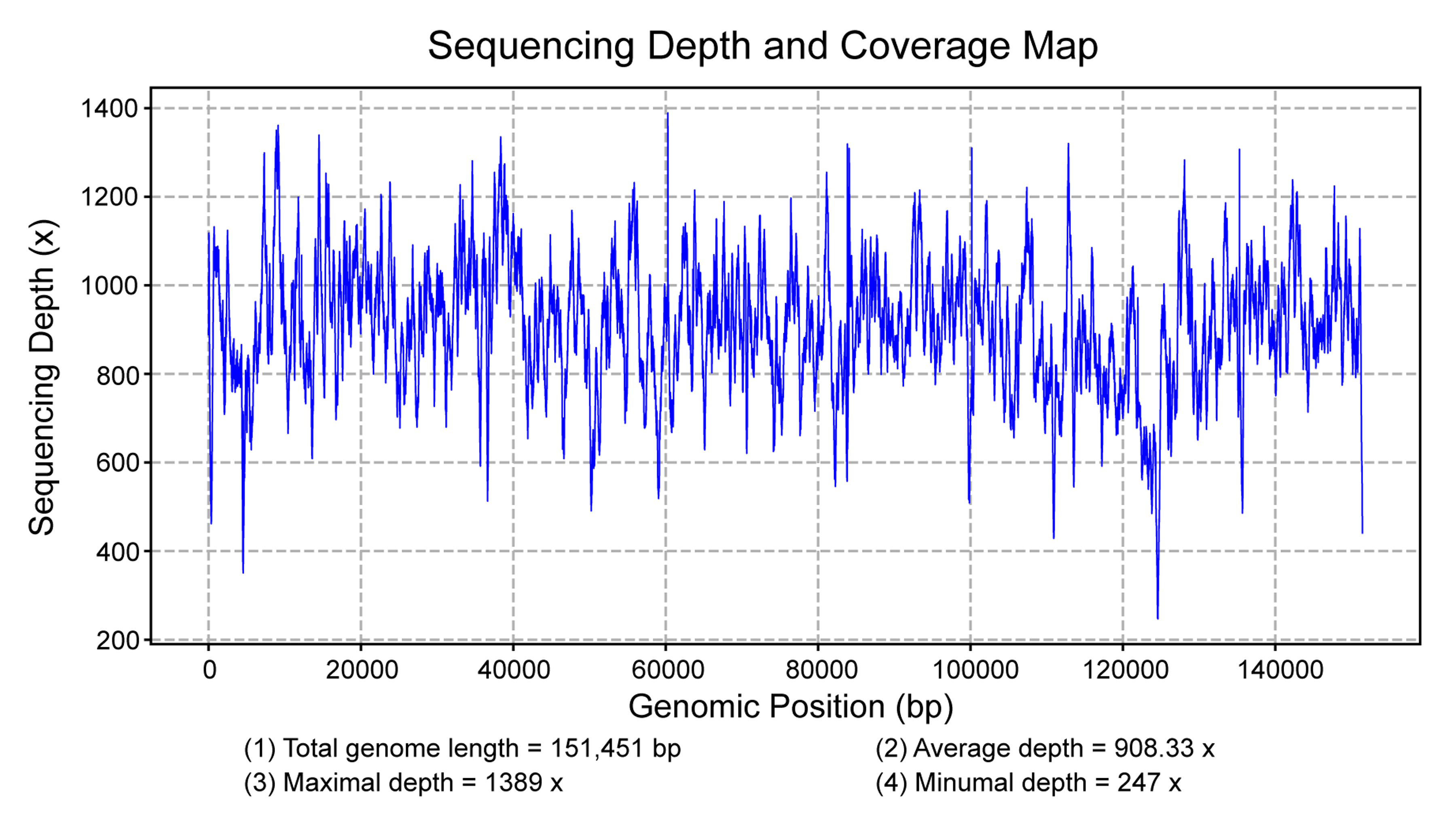

Supplement: SupplymentaryFigure_1 600dpi6inch.tif [file TMDN_A_2387258_SM4778.tif]
